# Psychosocial predictors of mental well-being in higher education students: a comprehensive approach

**DOI:** 10.1186/s12889-026-28111-8

**Published:** 2026-06-13

**Authors:** Pedro Alcântara da Silva, Carla Branco, Sibila Marques, João Limão

**Affiliations:** 1 Institute of Social Sciences, University of Lisbon, Lisbon, Portugal; 2 Centre for Psychological Research and Social Intervention (CIS-Iscte), ISCTE-University Institute of Lisbon, Lisbon, Portugal; 3 cE3c – Centre for Ecology, Evolution and Environmental Changes & CHANGE – Global Change and Sustainability Institute, Faculty of Sciences, University of Lisbon, Lisbon, Portugal

**Keywords:** Higher education, Students, Well-being, Health, Psychosocial factors

## Abstract

**Background:**

Time spent in tertiary education can be crucial in establishing practices and attitudes that last a lifetime. Although higher education institutions can be one of the main promoters of health, students seem to be a particularly vulnerable group, exposed to constant pressure for academic results and to various stimuli with consequences for their health. It is common to find psychological distress situations, with high levels of depression, anxiety, stress, and even suicidal ideation. Strategies for preventing and promoting health in higher education need to be based on research on predictors of student well-being. Due to the large number of factors that can have a significant impact on student well-being, it is essential to analyze all of these influences together and identify potential priorities for intervention. In this study, this is done by taking a holistic approach to the various factors at multiple levels of analysis. The aim was to test the differential role of multiple predictors of mental well-being on a representative sample of Portuguese university students, using seven factors: (i) sociodemographic; (ii) health status; (iii) lifestyle choices; (iv) life satisfaction and self-esteem; (v) academic-related aspects; (vi) relationships; and (vii) leisure and cultural activities.

**Methods:**

The research is based on a probabilistic stratified cluster sample representative of the student population of 30 Portuguese public higher education institutions for the 2021/2022 academic year (*n* = 9,611). Mental well-being was measured using the WHO-5 Well-being Index. A hierarchical multiple regression analysis with seven blocks of predictors was performed to test the differential role of sociodemographic, health status, lifestyle, life satisfaction and self-esteem, academic-related, relationship, and leisure and cultural activity factors. Multiple imputation (15 datasets) was used to handle missing data.

**Results:**

Findings indicate that health, lifestyle, self-esteem, and life satisfaction are most predictive of mental well-being among students, followed by sociodemographic, leisure and cultural activities, academics, and relations. Conversely, lower levels of mental well-being were associated with being female, being younger, and experiencing persistent pain. By taking these multiple factors into account, we can better promote mental health interventions for students in higher education settings.

**Conclusions:**

These findings highlight the need for comprehensive mental health promotion strategies in higher education settings. Interventions should prioritize improving self-esteem, sleep quality, and leisure time, while also addressing the specific needs of more vulnerable groups, such as female and younger students. By adopting a psychosocial approach that considers multiple levels of influence, higher education institutions can play a pivotal role in promoting student mental well-being.

**Supplementary Information:**

The online version contains supplementary material available at 10.1186/s12889-026-28111-8.

## Background

Contemporary challenges in our societies, like the pandemic, require an articulated and multi-sectorial response [1]. Universities are one of the most important players in this domain and may act as important public health promotors, putting our knowledge to practice and advising best ways to proceed [2]. UNESCO statistics in March 2023 showed that 235 million students are enrolled in universities around the world and that this number is still expected to increase in the next years. Hence, universities have a huge potential to touch the lives of millions of people that study or interact with these academic environments.

Unfortunately, despite this enormous potential, data so far has been showing a glooming picture and the deterioration in the levels of health and well-being within these institutions. Population’s mental health has been decreasing worldwide, with a rise of 13% in mental health conditions to 2017 [3]. Globally, 14% of young people and adolescents aged 10–19 years’ experience mental health conditions, most commonly depression, anxiety and behavioral disorders, while suicide is the fourth leading cause of death among ages 15–29 [3]. Although no data are currently available, this pattern has likely worsened due to COVID-19. In this context, university students seem to be a particularly vulnerable group. Studies conducted prior to the pandemic already showed consistent signs of poor mental health and psychological suffering in students. For instance, a study considering 21 countries all over the world [4] showed that one fifth of university students has 12-month DSM IV- CIDI disorders and only 16.4% of these students have received adequate treatment. This situation has been exacerbated in the aftermath of pandemics, with longitudinal studies showing significant increases in psychological distress among university students [5–7]. Compared with non-students, studies have also shown that university students are a particular risk group, presenting higher levels of depression, anxiety, stress and suicidal ideation during the pandemic [8]. In fact, a recent meta-analysis considering a total of 90,879 university students showed a 31.2% rate of depressive symptoms and 39.4% rate of anxiety symptoms following the pandemics [9].

This difficult context creates the need to understand in more detail the main predictors of student’s mental health to propose proper interventions. So far, several studies have already tried to explore this issue. However, they have often relied on the test of specific predictors and/or small sized and convenient samples, thus limiting the generalization of results. In the present study, our goal was to take a step forward and adopt a holistic approach of the predictors of university students’ mental health, considering the contribution of different factors at multiple levels of analysis. This ambitious goal was possible because these analyses were based on a large, nationally representative sample of university students in the Portuguese context. As far as we know this is the first time that such a comprehensive study has been conducted at the national level. Although this study focuses on the Portuguese university population, previous studies have shown similar trends in mental health among students from other countries [6, 10, 11].

In this work we were interested in exploring particularly the predictors of students’ mental well-being. The World Health Organization (WHO) broadly defines mental health as “a state of well-being that enables people to cope with the stresses of life, to realize their abilities, to learn well and work well, and to contribute to their communities” ([3], p. 8). This definition includes both positive functioning (or mental well-being) and mental illness. Hence, mental well-being may be defined as an overall positive mental state [12]. Mental health and well-being are related but distinct concepts. In fact, low levels of mental well-being can develop over time into a mental health condition, but this can be prevented through adequate intervention strategies. At the same time, it is also possible to enjoy periods of good well-being, even in the presence of mental illness [13]. From an intervention point of view, it is fundamental that universities understand this distinction in order to provide appropriate services. While specific interventions (e.g., counselling) are needed for those suffering from mental illness, more general, universal prevention and promotion strategies (e.g., online information) may be appropriate in cases of lower levels of well-being. Given the increased risk of ill health among university students, it is fundamental to intervene at the earliest possible stage and to address the key factors for promoting mental well-being.

### Predictors of mental well-being in students: from individual to social and cultural influences

Studies conducted within the university context have identified the need to address predictors of student’s mental health at different levels of analysis. For instance, Byrd and McKinney [14] showed that student’s mental health was affected by factors at the individual, interpersonal and institutional levels. In the same vein, in their review of the field, Sharp and Theiler [15] defined the need to examine sociodemographic and situational, academic and performance-related, as well as personality and individual difference factors in predicting student’s distress levels. In line with these and following recent recommendations [3], the present study adopted a psychosocial approach, considering predictors at different levels of analysis, from individual to social and cultural factors. This approach is consistent with ecological perspectives on well-being, which emphasize that individual outcomes are shaped by multiple, interacting personal and contextual domains [16]. It is also grounded in the idea that health promotion should go beyond investing in mental health services, adopting a preventive perspective that considers the role of psychological and community factors in promoting well-being.

At the individual level, several studies have shown a significant association between mental well-being and different types of factors such as sociodemographic variables, health status and lifestyle choices, global life satisfaction and self-esteem and academic perceptions and behaviors. For instance, previous studies have shown that being a female [10, 11, 17–19], younger [11, 18–24], having a lower subjective social status [21], and being displaced from home are risk factors for lower well-being levels [11, 22]. At the same time, homosexual orientation has also shown negative associations with well-being levels in some studies [18, 19]. Another variable that has been associated with lower levels of well-being is nationality, with studies revealing poorer outcomes for domestic students than for international students [22, 23].

Better health status [24] and lifestyle choices have also been associated with higher levels of well-being. For instance, previous chronic conditions [18, 19], receiving mental health services [19], exercising less [18, 19, 25, 26], poorer food availability [18], and addictive behaviors (i.e., alcohol consumption) [27] have been shown to be associated with lower well-being levels. In this regard, sleep quality is an important variable in studies of university students, showing stronger associations with well-being than other factors (e.g., physical exercise) [19, 28].

From a different angle, studies have shown that factors related to global life satisfaction and self-esteem [29] have also been associated with higher levels of mental well-being among university students. Considering that life satisfaction corresponds to a more cognitive evaluation of one’s own life [30], studies have shown a positive correlation between this variable and mental well-being (associated with a more evaluative dimension) [20].

Finally, still at the individual level, better academic perceptions and outcomes have also been consistently associated factors with higher student well-being [19, 31]. For instance, studies have shown that negative learning experiences [21] and concerns about completing their studies due to the impact of the pandemic were negatively associated with well-being [32].

At the social level and cultural level, studies have shown a consistent positive association of well-being with perceived support and positive relationships [6, 18, 24, 29]. Moreover, other studies have also examined the role of participating in various cultural and recreational activities as important predictors of student’s well-being levels. In particular, previous studies have found consistent associations of higher Internet and social network use with poor well-being [33] and positive associations with nature/contemplation activities [20].

Taken together, these studies suggest a vast array of factors that may have a significant impact on well-being. Given the number of possible influences, it is important to analyze them together to identify potential priority areas for intervention in this area.

### The present study

In sum, the present study aimed to test the differential role of multiple predictors of mental well-being among higher education students. We tested these effects for the first time in a large, nationally representative sample of higher education students and in the Portuguese context. Adopting a psychosocial perspective, our aim was to test the effect of the following factors: (i) sociodemographic (i.e., gender, age, nationality); (ii) health status (e.g., subjective health, satisfaction with physical shape, COVID’19 previous infection); (iii) lifestyle choices (e.g., exercise, diet, leisure); (iv) life satisfaction and self-esteem (e.g., satisfaction with standard of living); (v) academic-related aspects (e.g., perceived academic success, time management); (vi) relationships (e.g., affective/romantic, family, friends); and (vii) leisure and cultural activities (e.g., attending sports events, walking in nature, using social networks). The selection of these factors was based on the literature review and discussions with critical stakeholders involved in the project (i.e., university boards, public services with an interest in the youth field).

As far as we know, this is the first time that these factors have been tested together as predictors of mental well-being in higher education students. The final goal of this exercise was to identify priority areas for intervention with this target group and provide appropriate recommendations to actors in this field.

## Methods

### Participants and data collection

The sample consists of 9611 valid questionnaires for the set of universities, polytechnics, and non-integrated schools, with a sampling error that varies between a minimum of 3.6% and a maximum of 6.2% for a 95% confidence interval per higher education institution (sociodemographic information is in Table 1). The sampling error for the set of universities is 1.47% and for the set of polytechnics and non-integrated schools is 1.32%, with the global sample having an error of 0.98%, for the same confidence interval. The research was based on a questionnaire survey applied to a probabilistic stratified cluster sampling [34, 35], representative of the population of 283,542 bachelor’s, and master’s students of 30 public higher education institutions in Portugal for the academic year 2021/2022 (13 universities and 17 polytechnic higher education institutes and non-integrated schools). The initial sample design included all 35 public higher education institutions in Portugal. However, five institutions (two universities and three non-integrated schools) were unable to participate in the research due to time constraints or difficulties in administering the questionnaire, given its organizational and/or the academic course characteristics of these institutions (see Supporting Information for more information on sampling procedure and data collection).

**Table 1:** Descriptive statistics

Variables	*n*	%	Mean	SD	Minimum	Maximum
Mental well-being	9162		54.50	18.73	0	100
*(Sociodemographic)*						
Sex	9570					
Male	3430	35.8				
Female	6128	64				
“Other”	12	0.1				
Age	9563					
16–24	8473	88.6				
25–29	554	5.8				
> 30	536	5.6				
Sexual orientation	9111					
Heterosexual	7966	87.4				
Non-heterosexual	1145	12.6				
Degree level	9345					
1st cycle	7833	83.8				
2nd cycle	1512	16.2				
First year of the 1st cycle	9345					
Yes	2883	69.1				
Other years	6462	30.9				
Moved from residency	9476					
Yes	4954	52.3				
Not moved	4522	47.7				
Nationality	9458					
Portuguese	8784	92.9				
Foreign nationality	674	7.1				
Perception of social class	9412		5.17	1.48	0	10
*(State of health)*						
Perception of general health	9604		3.85	0.73	1	5
Satisfaction with physical shape	9471					
Yes, I’m satisfied	2194	23.2				
I usually don’t worry about it	1382	14.6				
No, I would like to improve it	5895	62.2				
Pain or relevant physical discomfort in the last 3 months	9203					
Yes	4704	51.1				
No	4499	48.9				
Chronic illness or health condition	8737					
Yes	2059	23.6				
No	6678	76.4				
Had COVID-19	9215					
Yes	3594	39				
No	5621	61				
Worried about getting (re)infected with COVID-19	9397		3.64	2.89	0	10
Worried about becoming seriously ill from COVID-19	9236		5.63	3.41	0	10
Go to the doctor regularly	9611					
Yes	4430	46.1				
No	5181	53.9				
Do psychotherapy	9611					
Yes	877	9.1				
No	8734	90.9				
*(Lifestyle)*						
Perception of a healthy lifestyle	9591		3.24	0.72	1	5
Satisfaction with planned and regular physical exercise	9331		4.16	2.91	0	10
Satisfaction with planned and regular leisure time and breaks	9274		5.55	2.43	0	10
Satisfaction with the quality of sleep	9414		5.21	2.57	0	10
Satisfaction with care about food	9343		5.57	2.19	0	10
Moderate alcohol consumption	9611					
Yes	4032	42				
No	5579	58				
*(Life satisfaction and self-esteem)*						
* Satisfaction with*:						
Standard of Living	9418		6.91	2.06	0	10
Personal Health	9373		6.61	2.07	0	10
Achieving in Life	9378		6.37	2.16	0	10
Personal Relationships	9360		6.87	2.15	0	10
Personal Safety	9261		7.16	2.08	0	10
Community-Connectedness	9345		6.79	2.23	0	10
Future security	9200		5.91	2.22	0	10
Self-esteem	8754		49.53	11.99	10	70
*(Academia)*						
Lifestyle after entering higher education (less healthy)	9605		3.32	0.92		
Academic success	8863					
Worse than expected	2918	32.9				
As expected	4451	50.2				
Better than expected	1494	16.9				
Balanced study pace	9611					
Yes	2638	27.4				
No	6973	72.6				
Time management						
Short term planning	9102		2.84	1.08	1	6
Attitudes to time	9108		3.21	1.03	1	6
Hope related to work	8026		20.98	4.43	6	30
*(Relationships)*						
In an affective/romantic relationship	9094					
Yes	4211	46.3				
No	4883	53.7				
Have a regular sexual partner	8997					
Yes	4396	48.9				
No	4601	51.1				
Have someone special who is there when they need it	9302		5.93	1.54	1	7
Can count on friends when things go wrong	9314		5.89	1.37	1	7
Can talk about problems with family	9291		5.48	1.75	1	7
*(Leisure and cultural activities)*						
Walk in nature	9297		3.24	0.94	1	5
Receiving friends at home or going to friends’ houses	9302		3.23	1.03	1	5
Attend live music concerts	9292		2.30	0.91	1	5
Go to sports shows	9291		2.16	1.08	1	5
Social networks (Facebook, Instagram, Twitter, etc.)	9199		3.03	1.13	0	5

The application of the self-administered questionnaire survey took place between April 4th and June 30th, 2022, with distribution and supervision by a team of interviewers trained for this purpose by the company GfK. A link to access the digital questionnaire (Qualtrics) was provided in the classroom for mobile phone, tablet, or PC, or, alternatively, a paper version, with an average completion time of 30 min.

The sampling process was carried out through a random selection method of Curricular Units (CU) from the two teaching cycles from exhaustive lists of all existing CUs provided by each of the 30 institutions that participated in the study, leading to the aforementioned probabilistic sampling by clusters in which the clusters are the CUs. Taking into account the total number of students enrolled in each higher education institution in the academic year under consideration, the number of students to be interviewed was calculated for a reference sample of 4% for each of them, randomly and sequentially selecting the necessary CUs until this number was reached in a timely manner during the fieldwork period, controlling for overlaps of classes, repetition of individuals, class scheduling management, non-response, among other factors that could cause bias, in order to guarantee the technical quality of the sample.

The option for this cluster sampling method was due to the impossibility of building a lower-level probabilistic sample. Probabilistic sampling at the student level would require access to the list of all bachelor’s and master’s students of all higher education institutions with their respective contacts and personal data, which would not be feasible given the EU General Data Protection Regulation 2016/679 to which universities, polytechnic institutes and non-integrated schools are bound. Therefore, the most precise level of analysis prior to the student, which is the CU, was adopted, with the higher education institutions ensuring the supply of lists with all the CUs operating in the second semester of the 2021/2022 academic year. In this process, the teaching staff of 865 CUs of the 30 institutions participating in the study were contacted, resulting in 518 pre-scheduled and 477 scheduled, for the effective realization of the survey in 452 CUs.

The data collection was supported by the rectorates of the universities and presidencies of the polytechnic institutes, and of the non-integrated schools, which constituted a network of interlocutors responsible for the articulation between each higher education institution participating in the study and the research team of the project. This allowed access to all the information necessary for the construction of the sample, as well as the dissemination of information about the ongoing research and the collaboration in the mobilization of teachers and students to fill in the questionnaire.

The study was approved by the Ethics Committee of the Institute of Social Sciences of the University of Lisbon. Whenever required, the questionnaire was submitted to the ethics committees of higher education institutions or evaluated by their representatives. The access to the questionnaire by participants was dependent on prior acceptance of informed consent, which consisted of an explanatory text about the objectives of the study and guarantees of anonymity, confidentiality and data protection.

This study was conducted in strict compliance with the ethical and deontological principles of research, with respect to the informed consent of the participants, as well as with respect to anonymity, confidentiality and data security at the various stages of the research (collection and processing of information). In accordance with EU Regulation 2016/679 and Portuguese Law 58/2019, only the data necessary for the study were collected, and it was processed and protected in accordance with applicable legislation.

### Measures

#### Mental well-being (outcome)

Mental well-being was measured with the WHO-5 Well-being Index [36, 37], a short positively worded 5 items related to the well-being over the last two weeks (e.g., “I have felt calm and relaxed”), rated from 5 - All of the time to 0 - No time at all. Items were summed up and the raw score, ranging from 0 to 25, was multiplied by 4 to give a final score, with 100 representing the higher score on mental well-being (α = 0,85).

*Sociodemographic* measures included: Sex (Male; Female; “Other”); Age (16–24; 25–29; >30); Sexual orientation (Heterosexual; Non-heterosexual); Degree level (1st cycle; 2nd cycle); First year of the 1st cycle (Yes; Other years); Moved from residency (Yes; Not moved); Nationality (Portuguese; Foreign nationality); and, Perception of social class (from 0 – Lower than people in general to 10 – Higher than people in general).

#### Health status

Several aspects related to health were measured (adopted from Silva et al. [38]) as follows: perception of general health (from 1 – Excellent to 5 – Very poor; reversed); Satisfaction with the physical shape (Yes; I’m satisfied; I usually don’t worry about it; No; I would like to improve it); Pain or relevant physical discomfort in the last 3 months (Yes; No); Chronic illness or health condition (Yes; No). Additionally, participants were asked if they had COVID-19 (Yes; No); they were worried about getting (re)infected with COVID-19; they were worried about becoming seriously ill from COVID-19 (both from 0 – Not at all worried to 10 – Very worried); they go to the doctor regularly (Yes; No); and, they do psychotherapy (Yes; No).

#### Lifestyle

Participants asked a single item on the general perception of a healthy lifestyle: “Do you consider your lifestyle to be.” from 1 – very healthy to 5 – Not healthy at all, which was reverse scored. Healthy lifestyle habits were measured with 4 items (adapted) [39, 40], from 0 – Strongly dissatisfied to 10 – Strongly satisfied: “In general, to what extent are you satisfied with (1) your planned and regular physical exercise routine; (2) Planned and regular leisure and break times; (3) The quality of your sleep; and (4) “Your care regarding nutrition”. Participants answered a question about whether they had or not moderate alcohol consumption (Yes; No).

#### Life satisfaction and self-esteem

Life satisfaction was measured with the Personal Well-being Index [41], including one item for seven dimensions, measured from 0 – Strongly dissatisfied to 10 – Strongly satisfied: Standard of Living; Personal Health; Achieving in Life; Personal Relationships; Personal Safety; Community-Connectedness; and, Future security. Self-esteem was measured with the Rosenberg Self-Esteem Scale [42] with 10 items (α = 0,88), from 1 – Strongly disagree to 7 – Strongly agree. Items were added together, varying in a range from 10 to 70, with higher scores representing higher levels of self-esteem.

#### Academics 

Participants were asked about their lifestyle after entering higher education with a single item: “Compared to the period before entering higher education, would you say that your lifestyle has become…” from 1 – Much healthy to 5 – Much less healthy; their academic success (Worse than expected; As expected; Better than expected); and, if they had a balanced study pace (Yes; No). Time management related to the study was measured with the Time Management Questionnaire [43, 44], with items ranging from 1 - Never to 6 - Always, which resulted in two dimensions: “Short-term planning” (7 items; α = 0,89) and “Attitudes to time” (3 items; α = 0,78; long-term planning was not used due to lower levels of reliability). Hope related to work [45, 46] was measured with 6 items, from 1 – Strongly disagree to 5 – Strongly Agree (items negatively worded were excluded). Items were added together, varying in a range from 6 to 30, with higher numbers representing higher levels of hope in the future related to work (α = 0,84).

#### Relationships 

Participants were asked if they were in an affective/romantic relationship (Yes, No); and, if they had a regular sexual partner (Yes; No). Relationships with family, friends, and a significant other, were measured with three items adapted from the Multidimensional Scale of Perceived Social Support [47], as follows: “There is a special person who is around when I am in need”; “I can count on friends when things go wrong”; and, “I can talk about my problems with family” (from 1- Strongly disagree to 7 – Strongly agree).

#### Leisure and cultural activities 

Measures on Leisure and Cultural activities included 5 items [38], as follows: “Please, indicate the frequency with which you usually engage in each of the following cultural and leisure activities:” (from 1 - Never to 5 - Always)*“*Walk in nature”; “Receiving friends at home or going to friends’ houses”; “Attend live music concerts”; “Go to sports shows”; and, “In the last month, on a typical day when engaging in the online activities listed below, please indicate how much time you dedicate to social networks (Facebook, Instagram, Twitter, etc.)”, from 0 – No time at all to 6–6 h or more.

### Analysis procedure

We performed a hierarchical multiple regression analysis, with multiple imputation, using SPSS, version 28. Hierarchical regression analysis was conducted to investigate predictors of higher education students’ mental well-being through a psychosocial approach. It was performed with the method enter and 7 blocks of predictors, in the following order: (1st ) sociodemographic, (2nd ) health status, (3rd ) lifestyle, (4th ) life satisfaction and self-esteem, (5th ) academics, (6th ) relationships, (7th ) leisure and cultural activities. The order of entry was theoretically defined and followed a psychosocial logic, moving from more distal background characteristics to health, lifestyle, subjective psychosocial resources, and then context-specific academic, relational, and leisure/cultural domains. This allowed us to estimate the incremental contribution of each block after accounting for previously entered predictors.

We used multiple imputation to handle missing data, as missing values ranged from nearly 0 to 11% of cases (see S1_Table in the Supporting Information), and deletion would have retained only 51% of the 9611 students. Missing data diagnostics suggested that the data were not missing completely at random and supported the use of a missing at random framework; therefore, multiple imputation was preferred over listwise deletion to reduce potential bias and retain statistical power [48, 49]. Multiple imputation was conducted using Fully Conditional Specification, also known as multivariate imputation by chained equations. The imputation model included all variables used in the analyses, including the dependent variable and dummy variables [50–53]. Fifteen imputed datasets were generated [54], and hierarchical regression analysis was performed on each dataset and then pooled according to “Rubin’s rule” [55]. As SPSS does not report standardized results for the pooled sample, variable values were standardized after multiple imputation and prior to regression analysis. Imputed results are presented (results of the final model for the original sample are included in S3_Table in the Supporting Information). We followed the recommendations to report multiple imputation of missing data in higher education research [56].

## Results

Respondents show a low positive level of mental well-being, with a mean of 54.5 (*SD* = 18.73) on a scale from 0 to 100 (see Table 1 for descriptive statistics for all variables). Yet, this could be considered a low level of mental well-being, considering that the mean of the WHO Well-being Index in Portugal in 2012 was 65.5, and a cut-off of < 50 is used to select cases with poor mental well-being and to screen for depression [36]. In addition, respondents report a positive (but not too high) level of perceived general health (*M* = 3.85; *SD* = 0.73), perceived healthy lifestyle (*M* = 3.24; *SD* = 0.72), and self-esteem (*M* = 49.53; *SD* = 11.99).

Hierarchical multiple regression was performed with 7 models (see Table 2; standardized results are shown). Multicollinearity diagnostics indicated no evidence of problematic collinearity among predictors, with tolerance values above 0.20 and VIF values below 5. After sociodemographic predictors were included in the model (1st model; *R*^2^= 7%; *F*_(10, 9600)_ = 71.718; *p* < .001), predictors related to heath status (2nd model; Δ*R*^2^= 14%; Δ*F*_(10, 9590)_ = 173.576; *p* < .001), lifestyle (3rd model; Δ*R*^2^= 14%; Δ*F*_(6, 9584)_ = 338.711; *p* < .001), and life satisfaction and self-esteem (4th model; Δ*R*^2^= 11%; Δ*F*_(8, 9576)_ = 248.201; *p* < .001) showed the largest incremental contributions, with Δ*R²* values of 14%, 14%, and 11%, respectively, corresponding to medium incremental effects according to Cohen’s *f²* (0.181, 0.212, and 0.207). The subsquent models of predictors related to academics (5th model; Δ*R*^2^= 1%; Δ*F*_(7, 9569)_ = 35.148; *p* < .001), relationships (6th model; Δ*R*^2^= 0.4%; Δ*F*_(5, 9564)_ = 14.199; *p* < .001), and leisure and cultural activities (7th model; Δ*R*^2^= 2%; Δ*F*_(5, 9559)_ = 71.300; *p* < .001) explained smaller additional proportions of variance, with incremental Cohen’s *f²* values ranging from 0.007 to 0.038. The final model explained approximately 50% of the variance in mental well-being. Furthermore, results regarding lifestyle, life satisfaction and self-esteem, academics, and relationships are consistent across all models after sociodemographic and health status predictors are entered. Following this, we focus on the results of the final model (model 7).

**Table 2:** Predictors of the mental well-being of higher education students (standardized estimates)

	model 1	model 2	model 3	model 4	model 5	model 6	model 7	
	Socio demographic	State of Health	Lifestyle	Life satisfaction and self-esteem	Academia	Relationships	Leisure & Cultural Activities	
*(Sociodemographic)*								
Sex (ref. Male)								
Female	-,161***	-,131***	-,103***	-,112***	-,126***	-,132***	-,137***	
“Other”	-,043***	-,028**	-,034***	-,014	-,014	-,013	-,014	
Age (ref. >30):								
16–24	-,034*	-,048***	-,109***	-,055***	-,040***	-,043***	-,051***	
25–29	-,007	-,007	-,040***	-,016	-,010	-,011	-,014	
Sexual orientation (ref. Heterosexual): Non heterosexual	-,111***	-,056***	-,032***	,003	,003	,009	,012	
Degree level (ref. 2nd cycle): 1st cycle	,013	,009	-,002	,006	,011	,011	,012	
First year of the 1st cycle (ref. Other years)	-,004	-,013	-,019*	-,010	-,008	-,006	-,005	
Moved from residency (ref. Not moved)	,005	,019*	,029***	,032***	,035***	,033***	,018*	
Foreign nationality (ref. Portuguese)	-,001	,004	,010	,011	,014	,017*	,024**	
Perception of social class	,151***	,099***	,062***	,016	,014	,014	,009	
*(State of Health)*								
Perception of general health		,221***	,101***	,035***	,034***	,032**	,027**	
Satisfaction with physical shape (ref. Yes, I’m satisfied)								
I usually don’t worry about it		-,062***	-,018	,000	,007	,008	,008	
No, I would like to improve it		-,202***	-,097***	-,027**	-,018	-,019	-,022*	
Pain or relevant physical discomfort in the last 3 months (ref. No)		-,130***	-,074***	-,051***	-,048***	-,047***	-,046***	
Chronic illness or health condition (ref. No)		-,025*	-,029**	-,016	-,017*	-,017	-,011	
Had COVID-19 (ref. Not had)		,015	,011	-,004	,000	-,002	-,010	
Worried about getting (re)infected with COVID-19		-,003	,000	,022*	,015	,014	,024**	
Worried about becoming seriously ill from COVID-19		,022	,001	-,022*	-,020*	-,022*	-,028**	
Go to the doctor regularly		,072***	,034***	,004	,000	-,003	-,013	
Do psychotherapy		-,057***	-,047***	-,015	-,016*	-,017*	-,019*	
*(Lifestyle)*								
Perception of a healthy lifestyle			,097***	,075***	,055***	,053***	,043***	
Satisfaction with planned and regular physical exercise			,022*	,032**	,018	,020*	,001	
Satisfaction with planned and regular leisure time and breaks			,170***	,107***	,109***	,107***	,089***	
Satisfaction with the quality of sleep			,255***	,183***	,173***	,171***	,169***	
Satisfaction with care about food			,036**	-,004	-,020	-,019	-,014	
Moderate alcohol consumption			,002	,000	-,003	-,003	,003	
*(Life satisfaction and self-esteem)*								
* Satisfaction with:*								
Standard of Living				-,005	,005	,000	,003	
Personal Health				,008	,017	,019	,023	
Achieving in Life				,073***	,032**	,033**	,032**	
Personal Relationships				,059***	,061***	,051***	,045***	
Personal Safety				,011	,017	,011	,022*	
Community-Connectedness				,064***	,066***	,051***	,026*	
Future security				,071***	,047***	,045***	,047***	
Self-esteem				,238***	,212***	,200***	,200***	
*(Academia)*								
Lifestyle after entering higher education (less healthy)					-,041***	-,042***	-,045***	
Academic success (ref. Better than expected):								
Worse than expected					-,034**	-,034**	-,037**	
As expected					-,008	-,009	-,009	
Balanced study pace (ref. No)					,015	,014	,015	
*Time management*:								
Short term planning					,042***	,040***	,025**	
Attitudes to time					,072***	,073***	,068***	
Hope related to work					,041***	,039***	,029**	
*(Relationships)*								
In an affective/romantic relationship (ref. No)						-,014	,002	
Have a regular sexual partner (ref. No)						-,001	-,024	
Have someone special who is there when they need it						,015	,011	
Can count on friends when things go wrong						,033***	,020*	
Can talk about problems with family						,050***	,044***	
*(Leisure and Cultural Activities)*								
Walk in nature							,082***	
Receiving friends at home or going to friends’ houses							,043***	
Attend live music concerts							,033***	
Go to sports shows							,059***	
Social networks (Facebook, Instagram, Twitter, etc.)							,004	
* R* ^2^	0.070	0.212	0.350	0.462	0.475	0.479	0.498	
* R*^2^ Adjusted	0.069	0.211	0.348	0.460	0.473	0.476	0.495	
Δ*R*^2^		0.143	0.138	0.111	0.013	0.004	0.019	
Δ*F*	71.718***	173.576***	338.711***	248.20***	35.148***	14.199***	71.300***	
Cohen’s *f²* for Δ*R²* ^a^	0.075	0.181	0.211	0.206	0.026	0.008	0.037	
n	9611							

Standardized coefficients are presented with p-value represented as ***< 0.001, **< 0.01, *< 0.05

Model fit indicators are the means of these indicators across all imputation datasets, as SPSS does not provide these estimates for the pooled sample

^a^Incremental Cohen’s *f²* was calculated as Δ*R²* /(1 − *R*^2^_fiull_), where Δ*R²* is the change in R² from the previous step, and *R*^2^_fiull_ refers to the total *R*^2^ of the model including the predictors entered at that step

In the (7th ) final model, among sociodemographic predictors, female gender (β = − 0.137, *p* < .001; compared to male) and age between 16 and 24 years (β = − 0.051, *p* < .001; compared to > 30 years) are negatively associated with mental well-being among higher education students (see S2_Table in the Supporting Information for detailed parameters of the final model). In turn, having moved from a permanent residency (β = 0.018, *p* = .021; compared to not having moved) and foreign nationality (β = 0.024, *p* = .002; compared to Portuguese nationality) are positively associated with mental well-being among higher education students.

Among the predictors of health status, perceived general health is associated with higher mental well-being (β = 0.027, *p* = .007), as is concern about (re)infection with COVID-19 (β = 0.024, *p* = .009). Dissatisfaction with physical shape (“no, I would like to improve it”; β = − 0.022, *p* = .020), pain or relevant physical discomfort in the last 3 months (β = − 0.046, *p* < .001), worrying about becoming seriously ill from COVID-19 (β = − 0.028, *p* = .003), and undergoing psychotherapy (β = − 0.019, *p* = .013) are negatively associated with mental well-being among higher education students.

Among the lifestyle predictors, the perception of a healthy lifestyle (β = 0.043, *p* < .001) is positively associated with mental well-being. Additionally, satisfaction with planned and regular leisure time (β = 0.089, *p* < .001) and satisfaction with sleep quality (β = 0.169, *p* < .001) are positively associated with mental well-being among higher education students.

Among the predictors of life satisfaction and self-esteem, satisfaction with life accomplishments (β = 0.032, *p* = .008), satisfaction with personal relationships (β = 0.045, *p* < .001), satisfaction with personal safety (β = 0.022, *p* = .041), satisfaction with community ties (β = 0.026, *p* = .023), and satisfaction with future security (β = 0.047, *p* < .001) are positively associated with mental well-being. In addition, self-esteem (β = 0.200, *p* < .001) is associated with higher mental well-being among students in higher education.

Regarding the academics predictors, having a less healthy lifestyle after entering a higher education institution (β = − 0.045, *p* < .001), and doing worse than expected academically (β = − 0.037, *p* = .002) are negatively associated with mental well-being, while short-term planning (β = 0.025, *p* = .004), attitudes toward time (β = 0.068, *p* < .001), and hope related to work (β = 0.029, *p* = .002) are positively associated with mental well-being among higher education students.

Regarding relationships, being able to count on friends when things go wrong (β = 0.020, *p* = .033) and being able to talk about problems with family (β = 0.044, *p* < .001) are associated with higher mental well-being among students in higher education.

Finally, across predictors of leisure and cultural activities, walking in nature (β = 0.082, *p* < .001), receiving friends at home or going to friend’s houses (β = 0.043, *p* < .001), attending live music concerts (β = 0.033, *p* < .001), and go to sports shows (β = 0.059, *p* < .001) are positively associated with mental well-being among higher education students.

In sum, in the final model, the largest positive standardized coefficients were observed for self-esteem (β = 0.200, *p* < .001) and satisfaction with sleep quality (β = 0.169, *p* < .001), while the largest negative standardized coefficient was observed for female gender (β = − 0.137, *p* < .001). These coeficients correspond to small effect sizes, with self-esteem, satisfaction with sleep quality, and female gender showing Cohen’s f² values between 0.03 and 0.04. Other statistically significant positive associations included satisfaction with planned and regular leisure time (β = 0.089, *p* < .001), walking in nature (β = 0.082, *p* < .001), and attitudes toward time (β = 0.068, *p* < .001). Statistically significant negative associations were also observed for being aged 16–24 years (β = − 0.051, *p* < .001; compared to > 30 years), pain or relevant physical discomfort in the last 3 months (β = − 0.046, *p* < .001), and a less healthy lifestyle after entering higher education (β = − 0.045, *p* < .001). These remaining individual effects were below the conventional threshold for a small effect.

## Discussion

Despite general improvements in health indicators (i.e., higher life expectancy and lower mortality), population mental health has been declining worldwide, with higher-education students representing a particular risk group [3, 8, 9]. In this study, we used a representative sample of higher education students to examine psychosocial predictors of students’ mental well-being. This approach included predictors at different levels of analysis - from individual to social and cultural factors, as follows: sociodemographic, health status, lifestyle, life satisfaction and self-esteem, academic-related aspects, relationships, and leisure and cultural activities. Overall, the findings suggest that mental well-being among higher education students is shaped by the cumulative contribution of several interrelated domains rather than by any single dominant predictor. At the block level, health status, lifestyle, and, self-esteem and life satisfaction made the largest incremental contributions to explained variance in mental well-being, followed by sociodemographic, leisure and cultural activities, academics, and relationships. This pattern supports a multidimensional understanding of students’ mental well-being, while also indicating that the unique effects of individual predictors should be interpreted cautiously.

When all factors were considered simultaneously, self-esteem and satisfaction with sleep quality showed the largest positive associations with mental well-being among higher education students. However, the individual effect sizes of these predictors were small, suggesting that they should be interpreted as the strongest statistical predictors in the final model rather than as dominant determinants of mental well-being. These findings are consistent with previous studies that have shown a strong association between self-esteem and mental well-being among university students [29]. Additionally, global life satisfaction is closely related to well-being among university students [29]. In the present study, a consistent pattern of positive associations was observed between several life satisfaction domains and mental well-being. Specifically, satisfaction with future security, personal relationships, life accomplishments, community connectedness, and personal safety were associated with higher mental well-being.

The relatively stronger association of sleep quality with mental well-being, compared with other lifestyle factors such as physical activity, has been reported in previous studies [19, 28]. Alongside sleep quality, satisfaction with leisure time and the perception of a generally healthy lifestyle were positively associated with mental well-being. Although previous studies have linked physical exercise [18, 25, 26], healthy eating [18], and alcohol consumption [27] to mental well-being, in the present study satisfaction with physical exercise and concern about food were not associated after accounting for academics and leisure activities in the model. Similarly, alcohol consumption was not associated with well-being.

Regarding health status, pain or relevant physical discomfort was negatively associated with mental well-being among higher education students, as were concern about becoming seriously ill from COVID-19, dissatisfaction with physical shape, and attending psychotherapy. On the other hand, a positive perception of general health was positively associated with mental well-being, as was concern about being infected with COVID-19. While fear of becoming seriously ill from COVID-19 may reflect a heightened and challenging threat perception, concern about being infected with COVID-19 may reflect a lower perceived threat or be accompanied by greater coping appraisals, such as self-efficacy, which have been linked to more preventive behaviors against COVID-19 [57–59] and, consequently, to higher mental well-being. These findings are somewhat consistent with previous studies showing that better health status [24] was linked with higher well-being, while receiving mental health services was associated with lower well-being [19]. As in previous studies [18, 19], chronic illness or health condition was negatively associated with mental well-being, but this association was no longer statistically significant after the inclusion of self-esteem and life satisfaction. In our model, pain or significant physical discomfort showed a more relevant association with mental health than chronic illness. Similarly, seeing a doctor regularly, which was positively associated with mental well-being in earlier models, was no longer statistically significant after the inclusion of self-esteem and life satisfaction.

Being female was negatively associated with mental well-being and showed the third-largest standardized coefficient in the final model, although its individual effect size was small. The importance of gender has been demonstrated in several studies [10, 11, 17–19, 21]. Among other sociodemographic variables, younger age (i.e., 16–24 years) was also negatively associated with mental well-being, as in previous studies [11, 20, 21]. In alignment with previous evidence in this domain [60], these results highlight the need to consider emerging adulthood as a special developmental period worthy of attention. In fact, emerging adulthood [61] carries several challenges related to identity formation and role adjustment that may make the transition to university especially challenging. At the same time, this is the period during which several psychiatric conditions may develop for the first time, making younger students more vulnerable when navigating the complex university context [62]. In a similar vein, several studies have shown that being a woman may represent an added risk for well-being [63]. Explanations for this are multifactorial in nature but may involve several factors, ranging from neurodevelopmental changes [63] to more psychological and social aspects related, for instance, to the use of emotional coping strategies [64] and to more stressors related to interpersonal relationships and societal expectations [65]. However, further studies are needed to better understand these age and gender differences in the well-being of university students. Importantly, this evidence highlights the need to develop specific interventions targeting the needs of these more vulnerable groups.

Moving away from permanent residence and foreign nationality were positively associated with mental well-being among higher education students. These results are consistent with previous studies showing better outcomes for international students [22, 23] compared to national students, but inconsistent with previous studies showing that being displaced from home was associated with lower levels of well-being [11, 22]. Although this finding contrasts with part of the previous literature, one possible interpretation is that moving away from home and being an international student may capture partly overlapping experiences. While being far away from home may be associated with increased vulnerability, it may also be associated with greater resilience and adaptive coping strategies [66]. Thus, the positive association observed among students who left home to study may reflect adaptive processes similar to those previously described among some international students. While previous research has shown that students with a homosexual orientation [18, 19] or lower social status [11, 22] have lower levels of mental well-being, in this study these associations were no longer statistically significant after including self-esteem and life satisfaction.

Among leisure activities, walking in nature was positively associated with mental well-being. Previous studies have shown a positive association between nature/contemplation activities and better well-being [20] and a negative association with social network use [25, 33]. In this study, the use of social networks was not associated with mental well-being, whereas other leisure and cultural activities were positively associated with it, including having friends over or visiting friends, attending sports events, and attending live music concerts.

Among the academic variables, attitudes toward time management were positively associated with mental well-being. Additionally, short-term planning and hope about future work were positively associated with mental well-being, while academic failure was associated with lower mental well-being. Relatedly, a less healthy lifestyle after entering higher education was negatively associated with mental well-being. Consistently, previous studies have shown that better academic perceptions and outcomes were positively associated with mental well-being among higher education students [19, 31]. Our findings suggest that maintaining a healthy lifestyle after entering higher education, as well as positive time management strategies, may be relevant factors to consider in initiatives aimed at supporting mental well-being among higher education students.

Relatedly, perceived support and positive relationships have been consistently associated with well-being [6, 7, 18, 24, 29]. In this study, being able to talk about problems with family and being able to count on friends when things go wrong were associated with higher mental well-being among higher education students.

Taken together, these findings suggest that interventions to promote the mental well-being of higher education students should adopt a multidimensional approach rather than focusing on isolated predictors. These interventions may benefit from addressing multiple interrelated domains, including self-esteem, life satisfaction, and lifestyle factors such as sleep quality and leisure activities, which, in turn, may also be connected to self-esteem and life satisfaction [67, 68]. Accordingly, interventions addressing leisure activities could consider nature activities, sports shows, and quality time with friends (i.e., having friends at home or going to friends’ houses). To promote positive academic experiences, interventions could address maintaining healthy lifestyles (especially after entering higher education), alongside broader lifestyle and leisure patterns, as well as time management strategies and hope for future work. In addition, interventions could be tailored to the needs of specific demographic groups, such as women and younger students. Other groups that may benefit from tailored interventions would be students who experience pain or relevant physical discomfort for a significant amount of time and those who are in psychotherapy, as these students may be experiencing greater vulnerability.

These findings contribute to informing strategies for the promotion of mental well-being among higher education students across several sectors, including policymakers, healthcare services, and universities. Higher education institutions could play a pivotal role in advancing mental health initiatives that benefit students and society. By bridging the gap between research and practical solutions, universities can become vital pillars in addressing the complex challenges surrounding mental well-being.

## Limitations and considerations for future studies

The sample used in this study had a significant amount of missing data, which is a common occurrence in representative samples. However, we believe we effectively mitigated this limitation by employing a method (i.e., multiple imputation) considered the most suitable for minimizing bias due to missing data [48].

It is important to note that this study cannot establish causality between the predictors and the outcome. Furthermore, our analysis focused on the direct associations between several predictors and the outcome, meaning that we did not examine potential indirect or mediational relationships among predictor variables (e.g., how lifestyle may influence life satisfaction or self-esteem). Although the psychosocial framework adopted in this study is compatible with such pathways, our primary aim was to examine the cumulative and incremental contribution of multiple theoretically relevant domains to mental well-being from a holistic perspective, rather than to test specific causal mechanisms. Future studies should examine these relationships using longitudinal designs, path analysis, experimental designs, or intervention outcomes.

Some variables included in the study were assessed using single-item indicators. Although single-item measures are frequently used in large-scale epidemiological and public health surveys due to their practicality and reduced respondent burden, they may provide less precision and reliability than multi-item scales. Therefore, future studies should consider using more comprehensive validated instruments whenever possible. Additionally, all variables were assessed using self-report measures collected at a single point in time, which may increase the possibility of common method variance (e.g., social desirability bias, negative affectivity, or shared method effects). However, the inclusion of conceptually distinct constructs, the large number of predictors examined simultaneously, and the differential pattern of associations observed (e.g., stronger associations for self-esteem and sleep quality compared to social network use) suggest that common method variance is unlikely to fully account for the findings. Future research could benefit from incorporating multiple assessment methods and data sources.

Relatedly, our findings include several effects with low Beta coefficients and small individual effect sizes, suggesting that the unique contribution of each predictor was limited when all variables were considered simultaneously. However, this pattern is not unexpected in a comprehensive model including multiple theoretically related predictors of mental well-being. In such models, variance is likely to be shared across several domains, meaning that individual coefficients may be small even when the predictors are conceptually relevant, and the model has substantial explanatory power. Consequently, lower coefficients should not be interpreted as evidence that these variables are irrelevant, but rather as reflecting the multidetermined nature of mental well-being and the complexity of the relationships among predictors. Moreover, the inclusion of these variables was grounded in previous research, supporting their theoretical relevance within a broader psychosocial framework.

Although we included several dimensions that predict mental well-being at different levels, such as individual and interpersonal, future studies (and interventions) should consider other levels of analysis, such as institutional (e.g., campus climate/tension [14]).

Despite these limitations, this study makes a significant contribution by providing a comprehensive examination of a wide range of predictors of mental well-being among higher education students, through a nationally representative sample. This comprehensive approach provides deeper insight into the factors affecting mental health in higher education contexts and could be used to promote targeted interventions to improve students’ mental well-being.

## Conclusions

This nationally representative study of Portuguese higher education students demonstrates that health status, lifestyle factors, life satisfaction, and self-esteem are the strongest psychosocial predictors of mental well-being, explaining a substantial portion of its variance. Self-esteem and sleep quality emerged as the most influential positive predictors, while female gender, younger age, and persistent physical pain were the strongest negative predictors.

These findings highlight the need for holistic, multi-level interventions in higher education. Universal strategies should prioritize self-esteem building, sleep hygiene education, promotion of healthy lifestyles (particularly during the transition to university), and increased opportunities for nature-based and social leisure activities. Targeted support should be directed towards female and younger students, as well as those experiencing chronic pain or already receiving psychotherapy. By fostering vibrant campus environments and time management resources, higher education institutions can play a pivotal role in promoting student mental well-being. Although focused on the Portuguese context, these findings are consistent with international evidence and can inform evidence-based approaches to address the growing mental health challenges faced by university students worldwide.

## Supplementary Information

Supplementary Material 1.

## Data Availability

The datasets used and/or analysed during the current study are available from the corresponding author on reasonable request.

## References

[1] The British Academy. The COVID Decade: Understanding the long-term societal impacts of COVID-19. Br Acad. 2021. 10.5871/bac19stf/9780856726583.001.

[2] Burns D, Dagnall N, Holt M. Assessing the impact of the COVID-19 pandemic on student wellbeing at universities in the United Kingdom: A conceptual analysis. Front Educ. 2020;5. 10.3389/feduc.2020.582882.

[3] World Mental Health Report. Transforming Mental Health for All. 1st ed. Geneva: World Health Organization; 2022. p. 1. 10.1002/wps.21018.

[4] Auerbach RP, Alonso J, Axinn WG, Cuijpers P, Ebert DD, Green JG, et al. Mental disorders among college students in the World Health Organization World Mental Health Surveys. Psychol Med. 2016;46(14):2955–70. 10.1017/S0033291716001665. 27484622 10.1017/S0033291716001665 PMC5129654

[5] Buizza C, Bazzoli L, Ghilardi A. Changes in college students mental health and lifestyle during the COVID-19 pandemic: A systematic review of longitudinal studies. Adolesc Res Rev. 2022;7(4):537–50. 10.1007/s40894-022-00192-7. 35966832 10.1007/s40894-022-00192-7 PMC9362152

[6] Conceição V, Rothes I, Gusmão R. The association between changes in the university educational setting and peer relationships: Effects in students’ depressive symptoms during the COVID-19 pandemic. Front Psychiatry. 2021;12:783776. 10.3389/fpsyt.2021.783776. 34970167 10.3389/fpsyt.2021.783776 PMC8712485

[7] Graupensperger S, Calhoun BH, Patrick ME, Lee CM. Longitudinal effects of COVID-19-related stressors on young adults’ mental health and wellbeing. Appl Psychol Health Well Being. 2022;14(3):734–56. 10.1111/aphw.12344. 35102692 10.1111/aphw.12344 PMC9746887

[8] Arsandaux J, Montagni I, Macalli M, Texier N, Pouriel M, Germain R, et al. Mental health condition of college students compared to non-students during COVID-19 lockdown: the CONFINS study. BMJ Open. 2021;11(8):e053231. 10.1136/bmjopen-2021-053231. 34413111 10.1136/bmjopen-2021-053231 PMC8380475

[9] Batra K, Sharma M, Batra R, Singh TP, Schvaneveldt N. Assessing the psychological impact of COVID-19 among college students: An evidence of 15 countries. Healthc (Basel). 2021;9(2):222. 10.3390/healthcare9020222. 10.3390/healthcare9020222 PMC7923198 33671363

[10] Campos R, Pinto V, Alves D, Rosa CP, Pereira H. Impact of COVID-19 on the mental health of medical students in Portugal. J Pers Med. 2021;11(10). 10.3390/jpm11100986. 10.3390/jpm11100986 PMC8540505 34683127

[11] Sequeira C, Araújo O, Lourenço T, Freitas O, Carvalho JC, Costa P. The impact of the COVID-19 pandemic on the mental health of Portuguese university students. Int J Ment Health Nurs. 2022;31(4):920–32. 10.1111/inm.12999. 35385603 10.1111/inm.12999 PMC9111582

[12] Cosma A, Költő A, Chzhen Y, Kleszczewska D, Kalman M, Martin G. Measurement invariance of the WHO-5 well-Being Index: Evidence from 15 European countries. Int J Environ Res Public Health. 2022;19(16):9798. 10.3390/ijerph19169798. 36011429 10.3390/ijerph19169798 PMC9407912

[13] Hewitt R. Measuring well-being in higher education. Oxford: HEPI Policy Note 13; 2019.

[14] Byrd DR, McKinney KJ. Individual, interpersonal, and institutional level factors associated with the mental health of college students. J Am Coll Health. 2012;60(3):185–93. 10.1080/07448481.2011.584334. 22420695 10.1080/07448481.2011.584334

[15] Sharp J, Theiler S. A review of psychological distress among university students: Pervasiveness, implications and potential points of intervention. Int J Adv Couns. 2018;40(3):193–212. 10.1007/s10447-018-9321-7.

[16] McLeroy KR, Bibeau D, Steckler A, Glanz K. An Ecological Perspective on Health Promotion Programs. Health Educ Q dezembro de. 1988;15(4):351–77. 10.1177/109019818801500401. 10.1177/109019818801500401 3068205

[17] Akhtar M, Kroener-Herwig B. Coping styles and Socio-demographic variables as predictors of psychological well-being among international students belonging to different cultures. Curr Psychol. 2019;38(3):618–26. 10.1007/s12144-017-9635-3.

[18] Kietrys D, Anderson E, Ray S. Self-perceived well-being among doctor of physical therapy students in the United States. J Wellness. 2023;5(1). 10.55504/2578-9333.1177.

[19] Ridner SL, Newton KS, Staten RR, Crawford TN, Hall LA. Predictors of well-being among college students. J Am Coll Health. 2016;64(2):116–24. 10.1080/07448481.2015.1085057. 26630580 10.1080/07448481.2015.1085057

[20] Büssing A, Rodrigues Recchia D, Hein R, Dienberg T. Perceived changes of specific attitudes, perceptions and behaviors during the Corona pandemic and their relation to wellbeing. Health Qual Life Outcomes. 2020;18(1):374. 10.1186/s12955-020-01623-6. 33256755 10.1186/s12955-020-01623-6 PMC7702679

[21] Dodd RH, Dadaczynski K, Okan O, McCaffery KJ, Pickles K. Psychological wellbeing and academic experience of university students in Australia during COVID-19. Int J Environ Res Public Health. 2021;18(3):866. 10.3390/ijerph18030866. 33498376 10.3390/ijerph18030866 PMC7908219

[22] Alharbi ES, Smith AP. Studying away and well-being: A comparison study between international and home students in the UK. Int Educ Stud. 2019;12(6):1. 10.5539/ies.v12n6p1.

[23] Preoteasa CT, Preoteasa E. Psychometric properties of romanian version of who-5 well-being index in dental students. Rom J Oral Rehabil. 2015;7(3):21–7.

[24] Liu C, McCabe M, Dawson A, Cyrzon C, Shankar S, Gerges N, et al. Identifying predictors of university students’ wellbeing during the COVID-19 pandemic-A data-driven approach. Int J Environ Res Public Health. 2021;18(13):6730. 10.3390/ijerph18136730. 34206579 10.3390/ijerph18136730 PMC8296899

[25] Islam MS, Akter R, Sikder MT, Griffiths MD. Weight-related status and associated predictors with psychological Well-being among first-year university students in Bangladesh: A pilot study. Int J Ment Health Addict. 2022;20(3):1354–69. 10.1007/s11469-020-00243-x.

[26] Pietsch S, Linder S, Jansen P. Well-being and its relationship with sports and physical activity of students during the coronavirus pandemic. Ger J Exerc Sport Res. 2022;52(1):50–7. 10.1007/s12662-021-00750-6. 40477338 10.1007/s12662-021-00750-6 PMC8456396

[27] Blank ML, Connor J, Gray A, Tustin K. Alcohol use, mental well-being, self-esteem and general self-efficacy among final-year university students. Soc Psychiatry Psychiatr Epidemiol. 2016;51(3):431–41. 10.1007/S00127-016-1183. X/METRICS PubMed PMID: 26831492. 26831492 10.1007/s00127-016-1183-x

[28] Kundayi Ravi R, Gamal M. Well-being among nursing students: Relationship between lifestyle behaviours, sleep quality and resilience. Afr J Nurs Midwifery. 2023;24(3). 10.25159/2520-5293/12619.

[29] Stallman HM, Ohan JL, Chiera B. The role of social support, being present and self-kindness in university student well-being. Br J Guid Counc. 2017;1–10. 10.1080/03069885.2017.1343458.

[30] Volkmer SA, Lermer E. Unhappy and addicted to your phone? -- Higher mobile phone use is associated with lower well-being. Comput Hum Behav. 2019;93:210–8. 10.1016/j.chb.2018.12.015.

[31] Gogoi M, Webb A, Pareek M, Bayliss CD, Gies L. University students’ mental health and well-being during the COVID-19 pandemic: Findings from the UniCoVac qualitative study. Int J Environ Res Public Health. 2022;19(15):9322. 10.3390/ijerph19159322. 35954680 10.3390/ijerph19159322 PMC9367732

[32] Plakhotnik MS, Volkova NV, Jiang C, Yahiaoui D, Pheiffer G, McKay K, et al. The perceived impact of COVID-19 on student well-being and the mediating role of the university support: Evidence from France, Germany, Russia, and the UK. Front Psychol. 2021;12:642689. 10.3389/fpsyg.2021.642689. 34322053 10.3389/fpsyg.2021.642689 PMC8311121

[33] Elbarazi I, Saddik B, Grivna M, Aziz F, Elsori D, Stip E, et al. The impact of the COVID-19 «infodemic» on well-being: A cross-sectional study. J Multidiscip Healthc. 2022;15:289–307. 10.2147/JMDH.S346930. 35228802 10.2147/JMDH.S346930 PMC8881924

[34] Arnab R. Survey Sampling Theory and Applications. London: Elsevier Science; 2017. 10.1016/B978-0-12-811848-1.00001-5.

[35] Lohr SL, Sampling. Design and Analysis. New York: Chapman and Hall/CRC; 2019. 10.1201/9780429296284.

[36] Topp CW, Østergaard SD, Søndergaard S, Bech P. The WHO-5 Well-Being Index: a systematic review of the literature. Psychother Psychosom. 2015;84(3):167–76. 10.1159/000376585. 25831962 10.1159/000376585

[37] WHO. Wellbeing Measures in Primary Health Care/ The Depcare Project. Report on a WHO Meeting. 1998. p. 45.

[38] Silva PA, Borrego R, Ferreira VS, Lavado E, Melo R, Rowland J, et al. Consumos e Estilos de Vida no Ensino Superior: O Caso dos Estudantes da ULisboa/2012. Lisboa: Colecção Estudos - SICAD/OPJ-ICS-ULisboa; 2015.

[39] Renpenning K, Taylor SG. Self-Care Science, Nursing Theory and Evidence-Based Practice. New York: Springer Publishing Company; 2011.

[40] Chambers R, Wakley G, Blenkinsopp A. Supporting Self Care in Primary Care. Oxford: Radcliffe Publishing; 2006.

[41] International Wellbeing Group. Personal Wellbeing Index: 5th Edition. Melbourne: Melbourne: Australian Centre on Quality of Life, Deakin University; 2013. pp. 1–41.

[42] Rosenberg M. Society and the adolescent self-image. Revised edition. Middletown: Wesleyan University; 1989.

[43] Britton BK, Tesser A. Effects of Time-Management Practices on College Grades. J Educ Psychol. 1991;83(3):405–10. 10.1037/0022-0663.83.3.405.

[44] Veiga F, Melim A. Portuguese adaptation of the academic time management questionnaire: new elements. Int J Dev Educational Psychol. 2010;4:343–52.

[45] Bryant FB, Harrison PR. Measures of hope and optimism: Assessing positive expectations of the future. Measures Personality Social Psychol Constructs. 2015;47–73. 10.1016/B978-0-12-386915-9.00003-6.

[46] Juntunen CL, Wettersten KB. Work hope: Development and initial validation of a measure. J Couns Psychol. 2006;53(1):94–106. 10.1037/0022-0167.53.1.94.

[47] Canty-Mitchell J, Zimet GD. Psychometric Properties of the Multidimensional Scale of Perceived Social Support in Urban Adolescents. Am J Community Psychol. 2000;28(3):391–400. 10.21865/RIDEP58.1.11. 10945123 10.1023/A:1005109522457

[48] Newman DA. Missing data. Organ Res Methods. 2014;17(4):372–411. 10.1177/1094428114548590.

[49] Schafer JL, Graham JW. Missing data: Our view of the state of the art. Psychol Methods. 2002;7(2):147–77. 10.1037/1082-989X.7.2.147. 12090408

[50] Gottschall AC, West SG, Enders CK. A comparison of item-level and scale-level multiple imputation for questionnaire batteries. Multivar Behav Res. 2012;47(1):1–25. 10.1080/00273171.2012.640589.

[51] Graham JW. Missing data analysis: making it work in the real world. Annu Rev Psychol. 2009;60(1):549–76. 10.1146/annurev.psych.58.110405.085530. 18652544 10.1146/annurev.psych.58.110405.085530

[52] Van Ginkel JR, Linting M, Rippe RCA, Van Der Voort A. Rebutting existing misconceptions about multiple imputation as a method for handling missing data. J Pers Assess. 2020;102(3):297–308. 10.1080/00223891.2018.1530680. 30657714 10.1080/00223891.2018.1530680

[53] Wu W, Jia F, Enders C. A comparison of imputation strategies for ordinal missing data on Likert scale variables. Multivar Behav Res. 2015;50(5):484–503. 10.1080/00273171.2015.1022644. 10.1080/00273171.2015.1022644 26610248

[54] Bodner TE. What Improves with Increased Missing Data Imputations? Struct Equation Modeling: Multidisciplinary J. 2008;15(4):651–75. 10.1080/10705510802339072.

[55] Rubin DB. Multiple Imputation for Nonresponse in Surveys. 1.^a^ ed. Wiley; 1987. (Wiley Series in Probability and Statistics). 10.1002/9780470316696.

[56] Manly CA, Wells RS. Reporting the use of multiple imputation for missing data in higher education research. Res High Educ. 2015;56(4):397–409. 10.1007/s11162-014-9344-9.

[57] Hedayati S, Damghanian H, Farhadinejad M, Rastgar AA. Meta-analysis on application of Protection Motivation Theory in preventive behaviors against COVID-19. Int J Disaster Risk Reduct. 2023;94. 10.1016/j.ijdrr.2023.103758. 10.1016/j.ijdrr.2023.103758 PMC10278899 37359108

[58] Machado BC, Pinto E, Silva M, Veiga E, Sá C, Kuhz S, et al. Impact of the COVID-19 pandemic on the mental and physical health and overall wellbeing of university students in Portugal. De Faria CMGM, editor. PLoS ONE. 2023;18(5):e0285317. 10.1371/journal.pone.0285317. 10.1371/journal.pone.0285317 PMC10159150 37141328

[59] Davis TEK, Sokan AE, Mannan A. Understanding the Impact of COVID-19 on Students in Institutions of Higher Education. HES. 2023;13(2):20. 10.5539/hes.v13n2p20.

[60] Krokstad S, Weiss DA, Krokstad MA, Rangul V, Kvaløy K, Ingul JM, et al. Divergent decennial trends in mental health according to age reveal poorer mental health for young people: repeated cross-sectional population-based surveys from the HUNT Study, Norway. BMJ Open. 2022;12(5):e057654. 10.1136/bmjopen-2021-057654. 35584877 10.1136/bmjopen-2021-057654 PMC9119156

[61] Arnett JJ. Emerging adulthood: A theory of development from the late teens through the twenties. Am Psychol. 2000;55(5):469–80. 10.1037/0003-066X.55.5.469. 10842426

[62] Solmi M, Radua J, Olivola M, Croce E, Soardo L, De Salazar G, et al. Age at onset of mental disorders worldwide: large-scale meta-analysis of 192 epidemiological studies. Mol Psychiatry. 2022;27(1):281–95. 10.1038/s41380-021-01161-7. 34079068 10.1038/s41380-021-01161-7 PMC8960395

[63] Salk RH, Hyde JS, Abramson LY. Gender differences in depression in representative national samples: Meta-analyses of diagnoses and symptoms. Psychol Bull. 2017;143(8):783–822. 10.1037/bul0000102. 28447828 10.1037/bul0000102 PMC5532074

[64] Matud M, Díaz A, Bethencourt J, Ibáñez I. Stress and Psychological Distress in Emerging Adulthood: A Gender Analysis. JCM. 2020;9(9):2859. 10.3390/jcm9092859. 32899622 10.3390/jcm9092859 PMC7564698

[65] Johansen R, Espetvedt MN, Lyshol H, Clench-Aas J, Myklestad I. Mental distress among young adults – gender differences in the role of social support. BMC Public Health. 2021;21(1):2152. 10.1186/s12889-021-12109-5. 34819040 10.1186/s12889-021-12109-5 PMC8611886

[66] Mostafa H, Lim Y. Examining the relationship between motivations and resilience in different international student groups attending US universities. J Int Stud. 2020;10(2):306–19. 10.32674/jis.v10i2.603.

[67] Maniaci G, La Cascia C, Giammanco A, Ferraro L, Palummo A, Saia GF, et al. The impact of healthy lifestyles on academic achievement among Italian adolescents. Curr Psychol. 2023;42(6):5055–61. 10.1007/s12144-021-01614-w.

[68] Wang F, Boros S. The relationship between physical activity, stress, life satisfaction and sleep quality. J Phys Educ Sport. 2019;19(34):227–34. 10.7752/jpes.2019.s1034.

