# Supplementary material for: Psychosocial predictors of mental well-being in higher education students: a comprehensive approach
Source: BMC Public Health. 2026 Jun 13;26:2394. doi: 10.1186/s12889-026-28111-8 (PMC13488587; doi:10.1186/s12889-026-28111-8)
Supplement: Supplementary file 1 [file 12889_2026_28111_MOESM1_ESM.docx]

# **Supporting information**

## **Data collection procedure**

The sampling process was carried out through a random selection method of Curricular Units (CU) from the two teaching cycles from exhaustive lists of all existing CUs provided by each of the 30 institutions that participated in the study, leading to the aforementioned probabilistic sampling by clusters in which the clusters are the CUs. Taking into account the total number of students enrolled in each higher education institution in the academic year under consideration, the number of students to be interviewed was calculated for a reference sample of 4% for each of them, randomly and sequentially selecting the necessary CUs until this number was reached in a timely manner during the fieldwork period, controlling for overlaps of classes, repetition of individuals, class scheduling management, non-response, among other factors that could cause bias, in order to guarantee the technical quality of the sample.

The option for this cluster sampling method was due to the impossibility of building a lower-level probabilistic sample. Probabilistic sampling at the student level would require access to the list of all bachelor’s and master’s students of all higher education institutions with their respective contacts and personal data, which would not be feasible given the EU General Data Protection Regulation 2016/679 to which universities, polytechnic institutes and non-integrated schools are bound. Therefore, the most precise level of analysis prior to the student, which is the CU, was adopted, with the higher education institutions ensuring the supply of lists with all the CUs operating in the second semester of the 2021/2022 academic year. In this process, the teaching staff of 865 CUs of the 30 institutions participating in the study were contacted, resulting in 518 pre-scheduled and 477 scheduled, for the effective realization of the survey in 452 CUs.

The data collection was supported by the rectorates of the universities and presidencies of the polytechnic institutes, and of the non-integrated schools, which constituted a network of interlocutors responsible for the articulation between each higher education institution participating in the study and the research team of the project. This allowed access to all the information necessary for the construction of the sample, as well as the dissemination of information about the ongoing research and the collaboration in the mobilization of teachers and students to fill in the questionnaire.

This study was conducted in strict compliance with the ethical and deontological principles of research, with respect to the informed consent of the participants, as well as with respect to anonymity, confidentiality and data security at the various stages of the research (collection and processing of information). In accordance with EU Regulation 2016/679 and Portuguese Law 58/2019, only the data necessary for the study were collected, and it was processed and protected in accordance with applicable legislation.

## **Descriptives**

Table S1. Descriptives and missing at the item-level.

|  | ***n*** | ***M*** | ***SD*** | ***Missing*** | |
| --- | --- | --- | --- | --- | --- |
|  |  |  |  | ***n*** | ***%*** |
| Mental well-being scale: | |  |  |  |  |
| Item 1 | 9349 | 2.79 | 1.21 | 262 | 2.7 |
| Item 2 | 9333 | 2.69 | 1.182 | 278 | 2.9 |
| Item 3 | 9321 | 2.21 | 1.294 | 290 | 3 |
| Item 4 | 9298 | 2.76 | 1.219 | 313 | 3.3 |
| Sex (ref. Male) |  |  |  |  |  |
| Female | 9570 |  |  | 41 | 0.4 |
| "Other" | 9570 |  |  | 41 | 0.4 |
| Age (ref. >30): |  |  |  |  |  |
| 16-24 | 9563 |  |  | 48 | 0.5 |
| 25-29 | 9563 |  |  | 48 | 0.5 |
| Sexual orientation (ref. Heterosexual): Not heterosexual | 9111 |  |  | 500 | 5.2 |
| Degree level (ref. 2nd cycle): 1st cycle | 9345 |  |  | 266 | 2.8 |
| First year of the 1st cycle (ref. Other years) | 9345 |  |  | 266 | 2.8 |
| Moved from family home (ref. Not moved) | 9476 |  |  | 135 | 1.4 |
| Foreign nationality (ref. Portuguese) | 9458 |  |  | 153 | 1.6 |
| Perception of social class | 9412 | 5.17 | 1.479 | 199 | 2.1 |
| Perception of general health | 9604 | 3.85 | 0.732 | 7 | 0.1 |
| Satisfaction with physical shape (ref. Yes, I'm satisfied) |  |  |  |  |  |
| I usually don't worry about it | 9471 |  |  | 140 | 1.5 |
| No, I would like to improve it | 9471 |  |  | 140 | 1.5 |
| Pain or relevant physical discomfort in the last 3 months (ref. No) | 9203 |  |  | 408 | 4.2 |
| Chronic illness or health condition (ref. No) | 8737 |  |  | 874 | 9.1 |
| Had COVID-19 (ref. Not had) | 9215 |  |  | 396 | 4.1 |
| Worried about getting (re)infected with COVID-19 | 9397 | 3.64 | 2.898 | 214 | 2.2 |
| Worried about becoming seriously ill from COVID-19 | 9236 | 5.63 | 3.415 | 375 | 3.9 |
| Go to the doctor regularly | 9611 |  |  | 0 | 0 |
| Do psychotherapy | 9611 |  |  | 0 | 0 |
| Perception of healthy lifestyle | 9591 | 3.24 | 0.72 | 20 | 0.2 |
| Satisfaction with planned and regular physical exercise | 9331 | 4.16 | 2.912 | 280 | 2.9 |
| Satisfaction with planned and regular leisure time and breaks | 9274 | 5.55 | 2.426 | 337 | 3.5 |
| Satisfaction with the quality of sleep | 9414 | 5.21 | 2.565 | 197 | 2 |
| Satisfaction with care in relation to food | 9343 | 5.57 | 2.187 | 268 | 2.8 |
| Moderate alcohol consumption | 9611 |  |  | 0 | 0 |
| *Satisfaction with:* |  |  |  |  |  |
| Standard of Living | 9418 | 6.91 | 2.056 | 193 | 2 |
| Personal Health | 9373 | 6.61 | 2.073 | 238 | 2.5 |
| Achieving in Life | 9378 | 6.37 | 2.162 | 233 | 2.4 |
| Personal Relationships | 9360 | 6.87 | 2.145 | 251 | 2.6 |
| Personal Safety | 9261 | 7.16 | 2.077 | 350 | 3.6 |
| Community-Connectedness | 9345 | 6.79 | 2.232 | 266 | 2.8 |
| Future security | 9200 | 5.91 | 2.219 | 411 | 4.3 |
| Self-esteem scale: |  |  |  |  |  |
| Item 1 | 9374 | 5.11 | 1.465 | 237 | 2.5 |
| Item 2 | 9292 | 4.15 | 2.036 | 319 | 3.3 |
| Item 3 | 9345 | 5.73 | 1.219 | 266 | 2.8 |
| Item 4 | 9323 | 5.49 | 1.379 | 288 | 3 |
| Item 5 | 9325 | 4.9 | 1.949 | 286 | 3 |
| Item 6 | 9301 | 4.69 | 2.018 | 310 | 3.2 |
| Item 7 | 9295 | 5.51 | 1.379 | 316 | 3.3 |
| Item 8 | 9200 | 3.62 | 2.135 | 411 | 4.3 |
| Item 9 | 9211 | 5.18 | 1.866 | 400 | 4.2 |
| Item 10 | 9239 | 5.2 | 1.512 | 372 | 3.9 |
| Lifestyle after entering higher education ( less healthy) | 9605 | 3.32 | 0.92 | 6 | 0.1 |
| Academic success (ref. Better than expected): |  |  |  |  |  |
| Worse than expected | 8863 |  |  | 748 | 7.8 |
| As expected | 8863 |  |  | 748 | 7.8 |
| Balanced study pace (ref. No) | 9611 |  |  | 0 | 0 |
| Time management scale: |  |  |  |  |  |
| Item 1 | 9054 | 3.39 | 1.145 | 557 | 5.8 |
| Item 2 | 9033 | 3.03 | 1.294 | 578 | 6 |
| Item 3 | 8993 | 3.45 | 1.128 | 618 | 6.4 |
| Item 4 | 8965 | 2.79 | 1.389 | 646 | 6.7 |
| Item 5 | 9014 | 3.04 | 1.437 | 597 | 6.2 |
| Item 6 | 9004 | 2.81 | 1.417 | 607 | 6.3 |
| Item 7 | 9018 | 2.77 | 1.524 | 593 | 6.2 |
| Item 8 | 9004 | 2.21 | 1.343 | 607 | 6.3 |
| Item 9 | 8914 | 2.65 | 1.409 | 697 | 7.3 |
| Item 10 | 8915 | 3.32 | 1.38 | 696 | 7.2 |
| Work hope scale: |  |  |  |  |  |
| Item 1 | 8920 | 4.03 | 0.983 | 691 | 7.2 |
| Item 2 | 8733 | 3.22 | 0.988 | 878 | 9.1 |
| Item 3 | 8722 | 3.88 | 0.94 | 889 | 9.2 |
| Item 4 | 8648 | 3.02 | 1.109 | 963 | 10 |
| Item 5 | 8623 | 3.49 | 0.975 | 988 | 10.3 |
| Item 6 | 8518 | 3.32 | 0.975 | 1093 | 11.4 |
| In a affective/romantic relationship (ref. No) | 9094 |  |  | 517 | 5.4 |
| Have a regular sexual partner (ref. No) | 8997 |  |  | 614 | 6.4 |
| Have someone special who is there when they need it | 9302 | 5.93 | 1.544 | 309 | 3.2 |
| Can count on friends when things go wrong | 9314 | 5.89 | 1.37 | 297 | 3.1 |
| Can talk about problems with family | 9291 | 5.48 | 1.754 | 320 | 3.3 |
| Walk in nature | 9297 | 3.24 | 0.942 | 314 | 3.3 |
| Receiving friends at home or going to friends' houses | 9302 | 3.23 | 1.025 | 309 | 3.2 |
| Attend live music concerts | 9292 | 2.3 | 0.912 | 319 | 3.3 |
| Go to sports shows | 9291 | 2.16 | 1.075 | 320 | 3.3 |
| Social networks (Facebook, Instagram, Twitter, etc.) | 9199 | 3.03 | 1.129 | 412 | 4.3 |

Table S2. Regression estimates of model 7 for the pooled sample.

|  |  |  |  |  |  | ***Fraction Missing Info.*** | ***Relative Increase Variance*** | ***Relative Efficiency*** |
| --- | --- | --- | --- | --- | --- | --- | --- | --- |
|  | ***B*** | ***S.E.*** | ***β*** | ***t*** | ***Sig.*** |  |  |  |
| (Constant) | -1.956 | 1.828 |  | -1.070 | 0.285 | 0.057 | 0.060 | 0.996 |
| *(Sociodemographics)* |  |  |  |  |  |  |  |  |
| Sex (ref. Male) |  |  |  |  |  |  |  |  |
| Female | -5.335 | 0.335 | -0.137 | -15.945 | <.001 | 0.050 | 0.052 | 0.997 |
| "Other" | -7.612 | 4.035 | -0.014 | -1.887 | 0.059 | 0.061 | 0.064 | 0.996 |
| Age (ref. >30): |  |  |  |  |  |  |  |  |
| 16-24 | -2.984 | 0.677 | -0.051 | -4.406 | <.001 | 0.047 | 0.049 | 0.997 |
| 25-29 | -1.150 | 0.839 | -0.014 | -1.371 | 0.171 | 0.037 | 0.038 | 0.998 |
| Sexual orientation (ref. Heterosexual): Not heterosexual | 0.653 | 0.445 | 0.012 | 1.468 | 0.142 | 0.042 | 0.043 | 0.997 |
| Degree level (ref. 2nd cycle): 1st cycle | 0.632 | 0.409 | 0.012 | 1.544 | 0.123 | 0.061 | 0.065 | 0.996 |
| First year of the 1st cycle (ref. Other years) | -0.201 | 0.318 | -0.005 | -0.634 | 0.526 | 0.033 | 0.033 | 0.998 |
| Moved from family home (ref. Not moved) | 0.663 | 0.288 | 0.018 | 2.300 | 0.021 | 0.035 | 0.036 | 0.998 |
| Foreign nationality (ref. Portuguese) | 1.736 | 0.572 | 0.024 | 3.033 | 0.002 | 0.071 | 0.076 | 0.995 |
| Perception of social class | 0.114 | 0.104 | 0.009 | 1.093 | 0.274 | 0.067 | 0.071 | 0.996 |
| *(State of health)* |  |  |  |  |  |  |  |  |
| Perception of general health | 0.693 | 0.255 | 0.027 | 2.720 | 0.007 | 0.046 | 0.048 | 0.997 |
| Satisfaction with physical shape (ref. Yes, I'm satisfied) |  |  |  |  |  |  |  |  |
| I usually don't worry about it | 0.403 | 0.480 | 0.008 | 0.840 | 0.401 | 0.031 | 0.032 | 0.998 |
| No, I would like to improve it | -0.870 | 0.375 | -0.022 | -2.318 | 0.020 | 0.044 | 0.046 | 0.997 |
| Pain or relevant physical discomfort in the last 3 months (ref. No) | -1.737 | 0.303 | -0.046 | -5.739 | <.001 | 0.050 | 0.052 | 0.997 |
| Chronic illness or health condition (ref. No) | -0.474 | 0.377 | -0.011 | -1.257 | 0.209 | 0.116 | 0.129 | 0.992 |
| Had COVID-19 (ref. Not had) | -0.398 | 0.295 | -0.010 | -1.351 | 0.177 | 0.060 | 0.063 | 0.996 |
| Worried about getting (re)infected with COVID-19 | 0.157 | 0.060 | 0.024 | 2.612 | 0.009 | 0.045 | 0.046 | 0.997 |
| Worried about becoming seriously ill from COVID-19 | -0.152 | 0.051 | -0.028 | -2.966 | 0.003 | 0.050 | 0.052 | 0.997 |
| Go to the doctor regularly | -0.493 | 0.296 | -0.013 | -1.669 | 0.095 | 0.045 | 0.047 | 0.997 |
| Do psychotherapy | -1.252 | 0.507 | -0.019 | -2.471 | 0.013 | 0.022 | 0.022 | 0.999 |
| *(Lifestyle)* |  |  |  |  |  |  |  |  |
| Perception of healthy lifestyle | 1.113 | 0.258 | 0.043 | 4.307 | <.001 | 0.027 | 0.027 | 0.998 |
| Satisfaction with planned and regular physical exercise | 0.008 | 0.063 | 0.001 | 0.127 | 0.899 | 0.085 | 0.092 | 0.994 |
| Satisfaction with planned and regular leisure time and breaks | 0.685 | 0.070 | 0.089 | 9.797 | <.001 | 0.036 | 0.038 | 0.998 |
| Satisfaction with the quality of sleep | 1.235 | 0.066 | 0.169 | 18.720 | <.001 | 0.033 | 0.034 | 0.998 |
| Satisfaction with care in relation to food | -0.121 | 0.084 | -0.014 | -1.427 | 0.154 | 0.044 | 0.045 | 0.997 |
| Moderate alcohol consumption | 0.101 | 0.285 | 0.003 | 0.353 | 0.724 | 0.027 | 0.027 | 0.998 |
| *(Life satisfaction and self-esteem)* |  |  |  |  |  |  |  |  |
| *Satisfaction with:* |  |  |  |  |  |  |  |  |
| Standard of Living | 0.029 | 0.098 | 0.003 | 0.300 | 0.764 | 0.057 | 0.060 | 0.996 |
| Personal Health | 0.209 | 0.108 | 0.023 | 1.930 | 0.054 | 0.023 | 0.023 | 0.998 |
| Achieving in Life | 0.276 | 0.104 | 0.032 | 2.654 | 0.008 | 0.053 | 0.055 | 0.997 |
| Personal Relationships | 0.389 | 0.101 | 0.045 | 3.862 | <.001 | 0.067 | 0.071 | 0.996 |
| Personal Safety | 0.194 | 0.095 | 0.022 | 2.044 | 0.041 | 0.066 | 0.070 | 0.996 |
| Community-Connectedness | 0.220 | 0.096 | 0.026 | 2.281 | 0.023 | 0.085 | 0.092 | 0.994 |
| Future security | 0.397 | 0.094 | 0.047 | 4.232 | <.001 | 0.074 | 0.079 | 0.995 |
| Self-esteem | 0.312 | 0.016 | 0.200 | 19.593 | <.001 | 0.046 | 0.048 | 0.997 |
| *(Academia)* |  |  |  |  |  |  |  |  |
| Lifestyle after entering higher education ( less healthy) | -0.922 | 0.168 | -0.045 | -5.484 | <.001 | 0.047 | 0.048 | 0.997 |
| Academic success (ref. Better than expected): |  |  |  |  |  |  |  |  |
| Worse than expected | -1.458 | 0.478 | -0.037 | -3.048 | 0.002 | 0.135 | 0.153 | 0.991 |
| As expected | -0.323 | 0.420 | -0.009 | -0.770 | 0.441 | 0.142 | 0.162 | 0.991 |
| Balanced study pace (ref. No) | 0.642 | 0.344 | 0.015 | 1.865 | 0.062 | 0.038 | 0.039 | 0.997 |
| *Time management:* |  |  |  |  |  |  |  |  |
| Short term planning | 0.429 | 0.150 | 0.025 | 2.860 | 0.004 | 0.031 | 0.032 | 0.998 |
| Attitudes to time | 1.246 | 0.182 | 0.068 | 6.847 | <.001 | 0.092 | 0.101 | 0.994 |
| Hope related to work | 0.122 | 0.039 | 0.029 | 3.108 | 0.002 | 0.084 | 0.091 | 0.994 |
| *(Relationships)* |  |  |  |  |  |  |  |  |
| In a affective/romantic relationship (ref. No) | 0.086 | 0.574 | 0.002 | 0.150 | 0.881 | 0.109 | 0.120 | 0.993 |
| Have a regular sexual partner (ref. No) | -0.910 | 0.564 | -0.024 | -1.614 | 0.107 | 0.104 | 0.114 | 0.993 |
| Have someone special who is there when they need it | 0.136 | 0.114 | 0.011 | 1.196 | 0.232 | 0.055 | 0.058 | 0.996 |
| Can count on friends when things go wrong | 0.274 | 0.129 | 0.020 | 2.128 | 0.033 | 0.040 | 0.042 | 0.997 |
| Can talk about problems with family | 0.475 | 0.095 | 0.044 | 4.995 | <.001 | 0.069 | 0.073 | 0.995 |
| *(Leisure and cultural activities)* |  |  |  |  |  |  |  |  |
| Walk in nature | 1.631 | 0.170 | 0.082 | 9.597 | <.001 | 0.045 | 0.047 | 0.997 |
| Receiving friends at home or going to friends' houses | 0.788 | 0.170 | 0.043 | 4.638 | <.001 | 0.047 | 0.049 | 0.997 |
| Attend live music concerts | 0.680 | 0.183 | 0.033 | 3.711 | <.001 | 0.046 | 0.048 | 0.997 |
| Go to sports shows | 1.024 | 0.156 | 0.059 | 6.585 | <.001 | 0.064 | 0.067 | 0.996 |
| Social networks (Facebook, Instagram, Twitter, etc.) | 0.066 | 0.137 | 0.004 | 0.484 | 0.628 | 0.058 | 0.061 | 0.996 |

Table S3. Regression estimates of model 7 for the original sample.

|  | ***B*** | ***S.E.*** | ***β*** | ***t*** | ***Sig.*** |
| --- | --- | --- | --- | --- | --- |
| (Constant) | -2.293 | 2.518 |  | -0.911 | 0.363 |
| *(Sociodemographics)* |  |  |  |  |  |
| Sex (ref. Male) |  |  |  |  |  |
| Female | -5.636 | 0.452 | -0.148 | -12.474 | <.001 |
| "Other" | -3.737 | 7.525 | -0.005 | -0.497 | 0.619 |
| Age (ref. >30): |  |  |  |  |  |
| 16-24 | -2.855 | 0.928 | -0.049 | -3.076 | 0.002 |
| 25-29 | -1.917 | 1.140 | -0.025 | -1.682 | 0.093 |
| Sexual orientation (ref. Heterosexual): Not heterosexual | 0.468 | 0.611 | 0.008 | 0.765 | 0.444 |
| Degree level (ref. 2nd cycle): 1st cycle | 1.028 | 0.529 | 0.021 | 1.944 | 0.052 |
| First year of the 1st cycle (ref. Other years) | -0.188 | 0.434 | -0.005 | -0.432 | 0.665 |
| Moved from family home (ref. Not moved) | 0.269 | 0.384 | 0.007 | 0.700 | 0.484 |
| Foreign nationality (ref. Portuguese) | 2.144 | 0.863 | 0.026 | 2.486 | 0.013 |
| Perception of social class | 0.185 | 0.144 | 0.014 | 1.288 | 0.198 |
| *(State of health)* |  |  |  |  |  |
| Perception of general health | 0.863 | 0.349 | 0.034 | 2.475 | 0.013 |
| Satisfaction with physical shape (ref. Yes, I'm satisfied) |  |  |  |  |  |
| I usually don't worry about it | 0.459 | 0.673 | 0.008 | 0.682 | 0.495 |
| No, I would like to improve it | -0.465 | 0.497 | -0.012 | -0.936 | 0.349 |
| Pain or relevant physical discomfort in the last 3 months (ref. No) | -1.595 | 0.400 | -0.044 | -3.984 | <.001 |
| Chronic illness or health condition (ref. No) | -0.561 | 0.484 | -0.013 | -1.159 | 0.246 |
| Had COVID-19 (ref. Not had) | -0.622 | 0.395 | -0.016 | -1.576 | 0.115 |
| Worried about getting (re)infected with COVID-19 | 0.123 | 0.081 | 0.019 | 1.523 | 0.128 |
| Worried about becoming seriously ill from COVID-19 | -0.168 | 0.068 | -0.031 | -2.473 | 0.013 |
| Go to the doctor regularly | -0.557 | 0.391 | -0.015 | -1.423 | 0.155 |
| Do psychotherapy | -1.073 | 0.667 | -0.017 | -1.609 | 0.108 |
| *(Lifestyle)* |  |  |  |  |  |
| Perception of healthy lifestyle | 0.839 | 0.363 | 0.032 | 2.309 | 0.021 |
| Satisfaction with planned and regular physical exercise | -0.018 | 0.083 | -0.003 | -0.212 | 0.832 |
| Satisfaction with planned and regular leisure time and breaks | 0.821 | 0.096 | 0.106 | 8.586 | <.001 |
| Satisfaction with the quality of sleep | 1.206 | 0.090 | 0.165 | 13.441 | <.001 |
| Satisfaction with care in relation to food | 0.068 | 0.116 | 0.008 | 0.587 | 0.557 |
| Moderate alcohol consumption | 0.114 | 0.380 | 0.003 | 0.301 | 0.764 |
| *(Life satisfaction and self-esteem)* |  |  |  |  |  |
| *Satisfaction with:* |  |  |  |  |  |
| Standard of Living | 0.140 | 0.134 | 0.015 | 1.042 | 0.297 |
| Personal Health | 0.181 | 0.152 | 0.020 | 1.193 | 0.233 |
| Achieving in Life | 0.160 | 0.142 | 0.018 | 1.130 | 0.259 |
| Personal Relationships | 0.421 | 0.137 | 0.048 | 3.068 | 0.002 |
| Personal Safety | 0.328 | 0.129 | 0.036 | 2.553 | 0.011 |
| Community-Connectedness | 0.057 | 0.126 | 0.007 | 0.450 | 0.653 |
| Future security | 0.214 | 0.125 | 0.025 | 1.708 | 0.088 |
| Self-esteem | 0.333 | 0.022 | 0.218 | 15.302 | <.001 |
| *(Academia)* |  |  |  |  |  |
| Lifestyle after entering higher education ( less healthy) | -0.896 | 0.225 | -0.045 | -3.975 | <.001 |
| Academic success (ref. Better than expected): |  |  |  |  |  |
| Worse than expected | -1.711 | 0.607 | -0.044 | -2.817 | 0.005 |
| As expected | -0.427 | 0.525 | -0.012 | -0.812 | 0.417 |
| Balanced study pace (ref. No) | 0.458 | 0.453 | 0.011 | 1.011 | 0.312 |
| *Time management:* |  |  |  |  |  |
| Short term planning | 0.258 | 0.205 | 0.015 | 1.262 | 0.207 |
| Attitudes to time | 1.298 | 0.235 | 0.074 | 5.514 | <.001 |
| Hope related to work | 0.086 | 0.053 | 0.020 | 1.629 | 0.103 |
| *(Relationships)* |  |  |  |  |  |
| In a affective/romantic relationship (ref. No) | 0.176 | 0.747 | 0.005 | 0.236 | 0.813 |
| Have a regular sexual partner (ref. No) | -0.865 | 0.735 | -0.024 | -1.177 | 0.239 |
| Have someone special who is there when they need it | 0.291 | 0.160 | 0.023 | 1.821 | 0.069 |
| Can count on friends when things go wrong | 0.433 | 0.183 | 0.030 | 2.369 | 0.018 |
| Can talk about problems with family | 0.389 | 0.128 | 0.036 | 3.041 | 0.002 |
| *(Leisure and cultural activities)* |  |  |  |  |  |
| Walk in nature | 1.550 | 0.229 | 0.078 | 6.764 | <.001 |
| Receiving friends at home or going to friends' houses | 0.767 | 0.231 | 0.042 | 3.322 | <.001 |
| Attend live music concerts | 0.609 | 0.244 | 0.030 | 2.497 | 0.013 |
| Go to sports shows | 0.772 | 0.204 | 0.046 | 3.794 | <.001 |
| Social networks (Facebook, Instagram, Twitter, etc.) | -0.040 | 0.189 | -0.002 | -0.210 | 0.834 |
